# Improving pre-service teachers’ AI competencies: a quasi experimental study

**DOI:** 10.3389/fpsyg.2025.1642465

**Published:** 2025-10-29

**Authors:** Xiaotian Han

**Affiliations:** Shanghai Normal University Tianhua College, Shanghai, China

**Keywords:** AI competency, pre-service teachers, AI courses, higher education, quantitative research

## Abstract

UNESCO’s sustainable development agenda underscores the critical need to equip pre-service teachers with artificial intelligence competencies, thereby enabling future generations to attain the global sustainable goal of quality education. This study aims to evaluate the perceptions concerning the AI competencies of pre-service teachers and investigates the influence of AI course helpfulness on the AI competency of pre-service teachers. A total of 79 pre-service teachers participated in AI courses, covering topics from foundational AI principles to interdisciplinary applications. Employing a quantitative correlational research design, the study assessed participants’ perceptions of the helpfulness of these AI courses, alongside their AI competencies, which were evaluated across six domains: AI awareness, basic AI knowledge, basic AI skills, problem-solving, AI teaching practice, and ethics and safety. These results show that the overall AI competency stood at a moderate level. Pre-service teachers have increased awareness of AI and ethics while they exhibit lower in problem-solving, basic AI skills, and AI teaching practice. Secondly, the findings showed that the role of well-structured AI courses enhancing the development of pre-service teachers’ AI-related capabilities is mostly supported. Further research is needed to determine the long-term effect of these AI training on classroom practices and teaching effectiveness.

## 1 Introduction

Artificial intelligence (AI) is one of the most transformative technological innovations developed during the last decades (Kim et al., 2022). It was first conceptualized in 1956 and emulated intelligent human behavior transpired through technologies such as machine learning, natural language processing, and neural networks (Hamet and Tremblay, 2017; McCarthy, 2007). UNESCO Institute for Information Technologies initiated the project “AI in Education: Change at the Speed of Learning,” focusing on data analytics, personalized learning, and machine learning, hence showing how important AI played a role in education (UNESCO IITE, 2020). Then, the UNESCO sustainable development agenda (UNESCO, 2020, 2021) also identified the need for future generations to be better prepared for challenges now appearing increasingly obvious with increased urgency and called for integrating AI into schools as part of the global sustainable goal of achieving quality education. In that respect, the integration of AI in education has been recognized as a valuable yet context-dependent tool for educators and students, with the potential to enhance learning experiences when supported by appropriate training and ethical safeguards (Sanusi et al., 2022).

However, there is a significant scarcity of empirical studies that define the necessary skills and competencies for pre-service teachers to incorporate AI into K-12 education (Ayanwale et al., 2022; Frimpong, 2022). Moreover, research examining pre-service teachers’ intentions and readiness to adopt AI remains limited, particularly in the Pacific-Asia region (Su et al., 2022; Tedre et al., 2020; vonWangenheim et al., 2020). The purpose of this study is to assess pre-service teachers’ perceptions of AI competencies and the impact of AI courses on these competencies, thereby facilitating informed decisions regarding the strategic integration of AI education into their curricula to optimize learning outcomes in the digital era. The specific research questions are:

Research question 1 (RQ1): Do pre-service teachers exhibit differences in AI competency before participating in AI courses (pre-test group) versus after attending the courses (post-test group)?
Research question 2 (RQ2): Is there an association between the perceived helpfulness of AI courses and teacher AI competency?
Research question 3 (RQ3): To what extent does the perceived helpfulness of AI courses influence pre-service teachers’ AI competencies?

The significance of the study were listed as follows: (1) Existing research on AI competencies in higher education is largely centered on Western contexts, thus creating a noticeable gap in literature from Asia. This research aims to bridge this gap by offering an essential Asian perspective; (2) By exploring avenues to enhance AI competencies, this study contributes to achieving national educational objectives. Its findings elucidate the mechanisms through which AI courses enhance the understanding of AI among pre-service teachers and confirm the viability of AI education in Chinese higher education institutions. These insights will provide a foundation for policymakers and educational authorities to devise strategies tailored to the demands of modern education; and (3) Investigating the impact of AI courses will further aid educators in identifying educational models that optimize teacher AI competency. By nurturing AI competencies in pre-service teachers, the study advocates for a resilient educational framework that supports enduring teaching practices adaptable to forthcoming technological advancements.

## 2 Literature review

### 2.1 Definition and components of AI competency

AI competency is basic in the sense that recent AI technologies continue to develop and change smart classrooms, transform learning systems, and enable personalized learning experiences; thus, it should be one of the competencies to be taken into consideration while assessing pre-service teachers who will form the future of educational systems (Ayanwale et al., 2024). From this point of view, AI competency is a broad concept since the dimensions that compose it and the structure cannot be set alike, which leads to many different interpretations of this concept.

While the terms AI literacy and AI competency are normally used interchangeably, they are quite different in what they mean. AI Literacy is defined as the power of applying and using AI technologies, communicating well with AI, and using AI as a tool in everyday life (Long and Magerko, 2020). In contrast, AI competency refers to the capacity to “integrate AI-based technologies, skills, knowledge, and complementary resources in a way that establishes a competitive advantage” (Mikalef et al., 2023, p.5). Scholars have posited that literacy involved what to know and what skills are needed while competency related to the ability to apply that knowledge effectively and proficiency (Chiu et al., 2024). Beyond literacy and competency, scholars have also highlighted the role of teachers in AI education— make contextually appropriate decisions when applying AI in complex teaching scenarios, drawing on deep experience and ethical reasoning (Ng, 2021). This resonates with the focus on higher-order judgment within competency frameworks, as expert judgment stands as the apex of applying competency in real-world settings. In light of these distinctions, teacher AI competency spans a wide range of knowledge, skills, and attitudes that educators need to effectively comprehend, evaluate, utilize, and ethically employ AI across diverse teaching contexts, both inside and outside the classroom (Ng et al., 2021). This viewpoint not only emphasizes the application of technology but also incorporates the values of humanism. As a result, the role of teachers is transitioning from being mere disseminators of knowledge to serving as guides, motivators, and companions for students, a shift that aligns with the notion of “digital humanism” (Hong and Han, 2023; UNESCO, 2022).

Since 2018, scholars, educators, and organizations have actively engaged in discussions regarding the core components of AI competency, leading to the development of various frameworks from different perspectives. The instrumental contribution, however, comes in the form of the Five Big Ideas framework, in Touretzky et al. (2019a,b), for the development of the K-12 student’s key-concept proficiency: perception, representation, reasoning, learning, natural interaction, and social impact. An influential effort to summarize the broad landscape, but again, the dominant concentration of this framework was toward AI knowledge and skills rather than core competence. Long and Magerko (2020) took a more multidsiciplinarian approach, reviewing more than two decades of literature to synthesize 17 AI competencies into five key domains: what AI is, what AI can do, how AI works, how AI should be used, and how people perceive AI. This framework thus described deeper explorations of AI for more nuances among a broadened population. The “what AI is” includes explaining what AI is, understanding intelligence, and the distinctions between interdisciplinary vs. narrow AI; “what AI can do” involves strengths and weaknesses, and envisioning future AI; “how AI works” addresses issues of representation, decision-making, explainability, and sensors; while “how AI should be used” and “how people perceive of AI” explore ethics and programmability, respectively. Thus, this synthesis provided a better understanding of the competencies related to AI, hence helping educational programs catch up with needs more precisely and in a sustainable manner. Ng (2021), on the aforementioned grounds, further develop the divisiveness between AI literacy and AI competence by defining four themes of AI literacy for teachers and educators: knowing and understanding AI, applying AI, evaluating and creating AI, and considering AI ethics. This distinction underlines the fact that while literacy involves basic knowledge and its application, competence involves higher-order judgment and morality. Such differentiation is paramount to designing educational programs because, other than teaching about AI, they have to prepare teachers for responsible and effective AI use. Further, Chiu et al. (2024) held similar views on the distinction between AI literacy and AI competencies. They highlighted that AI literacy focuses on an individual’s ability to understand and explain how AI technologies function, whereas AI competency emphasizes the confidence and capability to use AI technologies effectively and beneficially in various contexts. Thus, AI competency can be seen as a broader and more abstract term compared to AI literacy. Building on this distinction, they identified the primary domains of AI competency as: technology, impact, ethics, collaboration, and self-reflection. Although researchers have different viewpoints and areas of emphasis, they consistently identify key components of AI competency, including AI knowledge and skills, AI applications and problem-solving, ethics, and interdisciplinary approaches. Notably, existing frameworks lack standardized metrics for cross-cultural validation, with the majority of studies confined to Western contexts—this creates a critical empirical gap in terms of generalizability.

### 2.2 Integrating AI competencies with teacher education

Integrating AI competencies with teacher education, the European Commission (2018) proposed a Digital Education Action Plan – Action 8 tailored specifically for primary and secondary educators. The framework outlined key components, including professional engagement, empowering learners, digital resources, assessment, teaching and learning, and facilitating learners’ digital competence. Subsequently, the European Commission (2022a) updated its digital competence framework to incorporate AI-related skills, knowledge, and attitudes, as well as data-related competencies. This updated framework aligns with the EU’s Digital Education Action Plan (2021–2027) and supports the development of AI learning resources for educators and trainers (European Commission, 2022b,c). Similarly, Japan’s AI Utilization Guidelines that proposed in August 2019 aimed to cultivate elite talents in information technology and digital sciences while enhancing AI capabilities nationwide (OECD, 2019). Germany’s “AI Strategy” that proposed in November 2018 focused on promoting digital and AI literacy across all age groups—ranging from children to the elderly—to facilitate necessary adjustments in the labor market (European Commission, 2022d). In February 2019, the United States launched the American AI Initiative, which sought to establish an AI training system, nurture the next generation of AI researchers and users, offer STEM courses, and ensure that all American citizens could fully engage with AI (Parker, 2019). Scholars in South Africa proposed four key strategies to restructure AI competencies in teacher education as follows as: providing hands-on AI workshops for differentiated instructions, having online learning for scaffolding self-paced AI tools, sharing learning experience in AI education communities, and participating AI training (Tarisayi, 2024). These policies and actions emerges a clear pattern: AI education has firmly established its role as a critical element in cultivating 21st-century talent.

Similarly, China put forward an AI teacher competency framework in 2022 (National Institute of Education Sciences, 2022), which lists key elements such as AI understanding and awareness, basic AI knowledge, basic AI skills, problem-solving, AI teaching practice, and ethics and safety. AI understanding and awareness emphasizes grasping the fundamentals of AI and its societal implications, including recognizing the differences between human and AI intelligence, the importance of human-computer collaboration, and addressing ethical considerations. The focus is on promoting responsible AI use and fostering harmonious development between humans and AI, grounded in legal, ethical, and moral awareness. Basic AI knowledge includes understanding the development history and trends of AI, grasping fundamental concepts and problem-solving logic, and mastering principles and technologies in various AI domains; it also involves becoming proficient in analyzing and solving problems within common AI application scenarios, such as intelligent education, autonomous driving, and intelligent security. Basic AI skills focus on selecting appropriate AI tools for primary and secondary education and creating practical platforms. This includes understanding common AI teaching products and guiding students effectively in hands-on practice. Additionally, it emphasizes applying intelligent technologies to solve typical AI tasks and leading students in extracurricular scientific and technological activities related to AI. Problem-solving outlines three main components: identifying real-world problems suitable for AI solutions, establishing models using information technology, designing problem-solving strategies, managing resources, fostering students’ innovation abilities, and organizing teams to research educational problems using AI technologies in collaboration with teachers, students, and technical experts. AI teaching practice involves applying teaching design methods that correspond to AI curriculum standards and equipment representation, using flexible teaching activities that provide effective guidance to student learning, and assisting other teachers in incorporating the application of AI technologies into their practices. Ethics and safety refer to maintaining an appropriate moral perspective on AI, adhering to privacy laws, and addressing potential adverse effects and risks associated with AI applications. This may also encompass conducting ethical analyses, performing security assessments, and tackling emerging sociopolitical challenges, for instance, designing AI tools that avoid reinforcing existing educational inequalities, establishing guidelines for responsible use of AI surveillance in classrooms, and advocating for digital sovereignty to ensure educational data remains under local governance (UNESCO, 2022). These efforts align with global initiatives to embed ethics in AI education, as emphasized in UNESCO’s AI Competency Framework for educators.

According to UNESCO (2024), the latest AI Competency Framework for educators stresses continuous professional development of teachers through which educators will make responsible and efficient use of AI while being able to mitigate potential risks toward students and society. Key elements of this framework include a human-centered approach, AI ethics, foundational AI knowledge and applications, AI pedagogy, and the use of AI for professional development. However, there is a lack of empirical research on how these frameworks adapt to non-Western educational systems, particularly in the Asia-Pacific region, where cultural and curricular contexts differ significantly.

### 2.3 Challenges of improving teacher AI competencies

Enhancing AI competence among teachers remains a significant challenge (Chiu et al., 2024). A primary obstacle is the insufficient AI-related knowledge and skills among both experienced and pre-service teachers (Huang, 2021). Many educators remained unsure about the influence of AI on teaching and learning (Carvalho et al., 2022). Research indicated that the majority of teachers were not trained to teach AI or integrate AI into their curricula during their undergraduate education, as specialized teacher training programs focusing on AI integration are still lacking globally (Frimpong, 2022; Su et al., 2022). While prior studies have introduced AI curricula, tools, and platforms into K-12 classrooms and highlighted their potential impact (i.e., Ng et al., 2021; Wong et al., 2020), teachers continue to struggle with determining which content to teach and selecting the appropriate pedagogical methods, tools, and platforms (Su et al., 2022). Consequently, they encounter challenges in grasping complex and abstract AI concepts, such as machine learning, deep learning, artificial neural networks, and natural language processing, as well as acquiring AI-related skills such as programming, data modeling, and analytics. Teachers also face notable pedagogical difficulties in facilitating interactions between students and AI in classrooms that have traditionally been dominated by human-to-human (teacher-student) engagement (Gunkel, 2012). As a result, both teachers and students encounter obstacles such as misconceptions about AI, misinformation, limitations, and underlying ethical concerns (Akgun and Greenhow, 2021; Hayes and Kraemer, 2017; Long and Magerko, 2020). Due to these challenges, many educators have voiced concerns and expressed hesitations about implementing AI education in their classrooms (Huang, 2021; Lin and Van Brummelen, 2021).

The infusion of AI technologies into educational curricula faces significant hurdles, primarily due to the sparse availability of professional development opportunities that equip educators with essential AI skills and pedagogical knowledge (Ayanwale et al., 2022; Han, 2021), who collectively emphasize the gap in training that prevents teachers from fully integrating AI into their teaching practices. Professional development in this context is aimed at enhancing the competencies, skills, and expertise of educators (OECD, 2009). Despite these initiatives, a notable number of teachers report a lack of confidence in AI instruction, attributing this to inadequate access to necessary technological pedagogical content knowledge, thereby indicating a shortfall in teacher AI competencies. The deficiency of computer science educators in K-12 education underscores the urgent need for robust training programs that not only enhance the educational capabilities of teachers but also modernize their knowledge to support AI teaching (Su et al., 2022), which reveal the scarcity of both in-service and pre-service professional development opportunities necessary for cultivating the requisite AI knowledge, skills, and attitudes (Frimpong, 2022; Mike and Rosenberg-Kima, 2021). Ng (2021) advocate for AI training programs that merge theoretical knowledge with practical technical support to foster an AI-centric mindset in educators (Lee and Perret, 2022) and co-design of learning resources (Lin and Van Brummelen, 2021), which aim to prepare teachers through collaborative resource design and professional development programs, a significant barrier remains the teachers’ own perception of their AI knowledge inadequacy (Ayanwale et al., 2022). Moreover, teachers’ attitudes toward AI and their confidence in using such technologies are critical predictors of their readiness to implement AI-driven instruction (Ayanwale et al., 2022). Therefore, it is crucial for educators to possess sufficient time and opportunities to promote the use of technology within the educational process, enhance their digital teaching skills, and intensify their commitment to empowering students within a digital learning context (Alfalah, 2018; Lin et al., 2023).

Moreover, there exists a profound lack of empirical studies that clearly define the necessary skills and competencies for teachers to successfully incorporate AI technology into K-12 education. Specifically, there is a dearth of research on teachers’ intentions and readiness to adopt AI in school environments (Ayanwale et al., 2022). Research into data literacy for teaching is also limited, with most studies focusing on the creation of assessment scales (Shreiner and Dykes, 2021; Trantham et al., 2021). Most research work related to AI curriculum is being conducted within North American and European nations, such as Finland and Spain. However, investigations about AI learning design and activities within the Asia-Pacific region remain sporadic (Su et al., 2022; Tedre et al., 2020; vonWangenheim et al., 2020). Therefore, educators should not be afraid to adopt proper AI-related technologies, such as adaptive learning systems, which will enhance their teaching methodology and make learning more personalized while at the same time enabling them to understand more precisely the academic progress and needs of the students. This can be well-voiced by Xu (2020): “teachers whose skills in using AI are poor may well be replaced by those whose skills are pretty good, for AI will make it possible to enhance the role of educators and promote a transformative change in their role of managing and making efficient decisions” (p. 290). Educators should also motivate students to use artificial intelligence-enhanced learning tools, like intelligent tutors and adaptive learning systems, for personalized support in learning-through self-diagnosis, automatic feedback, and encouragement of online collaboration among students (Cavalcanti et al., 2021). Although many curricula and other materials have been developed by researchers to bring forth the concept of AI, there is still a big difference between how teachers themselves actually train students in AI (Sanusi et al., 2024). This empirical gap is further exacerbated by a paucity of longitudinal studies tracking the development of AI competencies over time in real teaching contexts, leaving the long-term impact of training programs unclear.

Pre-service teachers form the bedrock of future educational systems. It is essential for policymakers and educational institutions to prioritize the development of AI competencies among pre-service teachers to meet the requirements of future AI-driven educational landscapes. Accordingly, China’s Ministry of Education has set specific targets to enhance these competencies to meet the needs of the 21st century (State Council of the PRC, 2019). The ministry’s policy underlines the necessity for sustainable educational strategies that prepare educators for a world characterized by rapid technological change and global workforce demands. As of 2024, over three hundred higher education institutions have introduced AI courses for undergraduates, encompassing both general and specialized AI-focused subjects. Nonetheless, the assessment of the quality of these AI courses is notably scant. Therefore, empirical research is urgently required to evaluate the efficacy of these courses, particularly through comparative studies that connect course design to measurable AI competency outcomes among pre-service teachers.

## 3 Methodology

This study adopted a quasi experimental design, collecting survey data from pre-service teachers to evaluate the stipulated research hypotheses. These pre-service teachers submitted information regarding their demographics (including gender, subjects instructed, and AI courses attended), their evaluations of AI application course benefits, and their perceived AI proficiency. This study did not engage in longitudinal data collection, which is typically more conducive to drawing causal conclusions.

### 3.1 Participants

**FIGURE 1:**
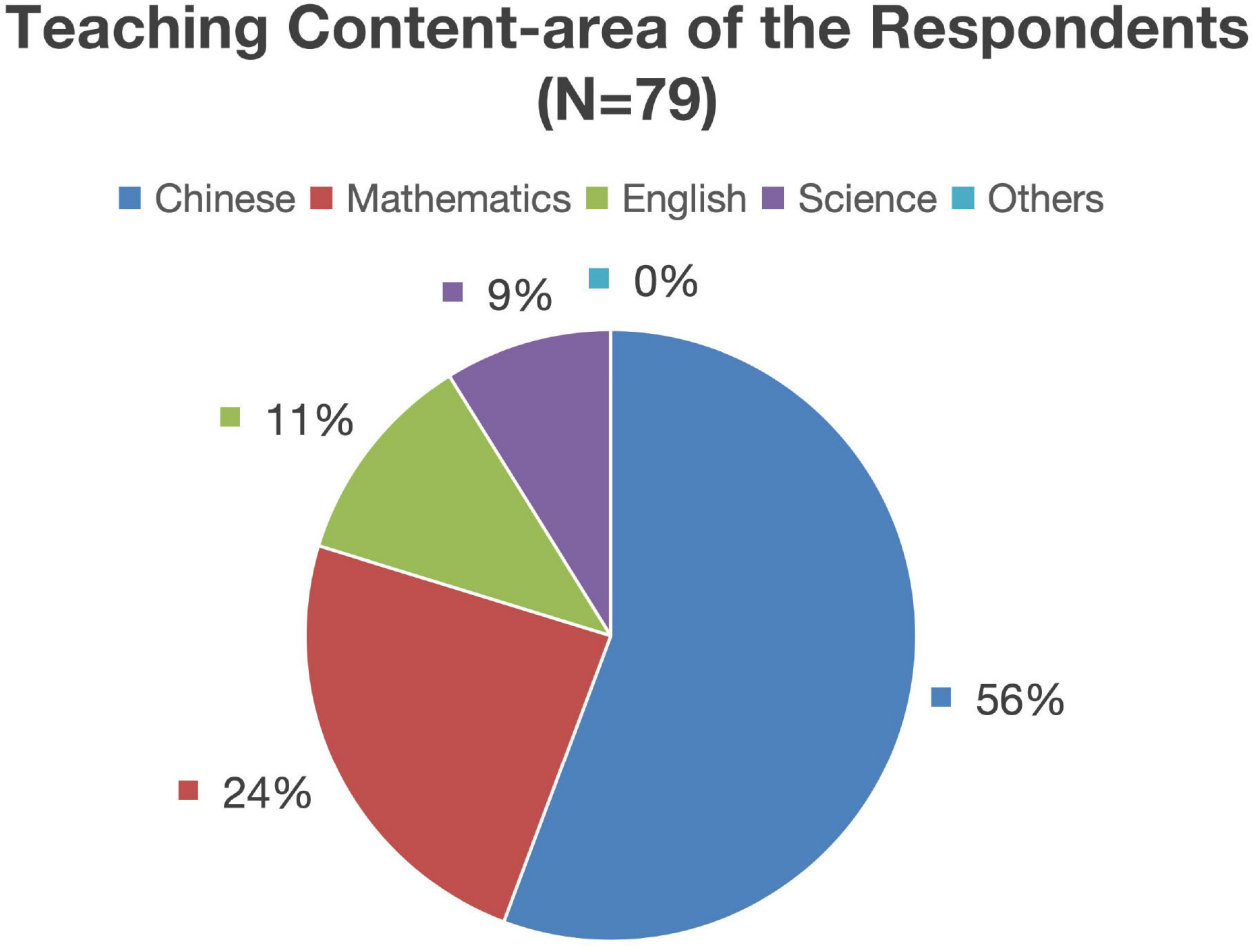
Teaching content-area of the respondents (*N* = 79).

The cohort comprised 79 pre-service teachers aged 19–21 from the School of Primary Education at a conventional university in Shanghai, China. Data collection spanned from February 2023 to June 2024, encompassing the Spring 2023, Fall 2023, and Spring 2024 semesters. The demographic details of the participants are summarized in Figure 1 and Table 1, which shows a gender distribution of 82.2% females and 17.8% males. Regarding teaching content area, about 67.1% of the participants were engaged in language arts (Chinese or English language arts), and 32.9% in mathematics or science.

**TABLE 1:** Demographics of the respondents (*N* = 79).

Variables	Gender	Frequency	Percentage
Gender	Male	14	17.8%
	Female	65	82.2%

The use of small-size sampling for participant recruitment, as suggested by McMillan and Schumacher (2010), may restrict the generalizability of the findings and elevate the risk of type II errors. Nevertheless, it offers valuable insights into the effectiveness of AI application courses. Future research involving larger, more diverse samples (e.g., across different regions or school types) is needed to validate these results. To mitigate potential biases, several measures were implemented during data collection. First, all participants provided informed consent following approval by the Institutional Review Board (IRB), ensuring compliance with ethical standards for human subjects research. Second, data were de-identified and stored anonymously, with all personal information removed from surveys and interview transcripts to safeguard participant privacy.

### 3.2 AI courses context

The study provided pre-service teachers with an introductory framework to the intelligent era, covering introduction to AI technology, AI programming language, AI education technology, teaching AI for primary school students, and interdisciplinary teaching applications of AI for primary school students. Participants were encouraged to consider AI from an interdisciplinary perspective, focusing on ethics, governance, and the impact of AI on societal progress. The course descriptions and the academic terms in which they were available are specified in Table 2.

**TABLE 2:** Descriptions of five AI courses.

Semester	Name	AI course descriptions
Spring 2023	Introduction to AI technology	This course provides an overview of fundamental definitions, the historical development, current trends in enterprises and industries within the AI field, and commonly used technical principles and applications. Topics covered include machine learning, knowledge graphs, computer vision, speech recognition, and natural language processing, enriched with practical case studies such as face recognition using Python.
Fall 2023	AI programming language	Students are introduced to programming languages like Python and Scratch, as well as other AI programming tools prevalent in primary education. The course aims to deepen understanding of AI’s foundational principles and applications while honing programming skills. Students engage in practical exercises that enhance their ability to use these languages for real-world applications, preparing them to flexibly deploy AI in future educational settings.
Fall 2023	AI education technology	This course instructs students on utilizing AI tools to design and develop micro-courses, fostering collaborative practices and enabling the presentation and exchange of their creations.
Spring 2024	Teaching AI for primary school students	Focused on delivering AI education to young learners, this course explores AI textbooks, applies experimental AI concepts in classrooms, interprets AI curricula, and integrates graphical/Python programming with intelligent robotics. It further involves the use of open-source hardware for AI applications, programming challenges, and studies on AI literacy among primary school pupils.
Spring 2024	Interdisciplinary teaching applications of AI for primary school students	This course merges AI technology with various disciplines such as Chinese, English, mathematics, history, biology, and physical education. Pre-service teachers are trained to view AI through the prism of subject-specific contexts, apply AI in resolving disciplinary issues, and develop skills for crafting comprehensive interdisciplinary AI curricula.

### 3.3 Instruments

#### 3.3.1 Pre-service teachers’ AI competency scale

The AI competency framework for pre-service teachers used in this study was adapted from the AI Ability Standards for Primary and Secondary School Teachers in China (National Institute of Education Sciences, 2022). The framework includes six subscales: AI understanding and awareness, basic AI knowledge, basic AI skills, problem solving, AI teaching practice, and ethics and safety. Each subscale comprises three items. The instrument utilized a five-point Likert scale (5-Strongly Agree, 4-Agree, 3-Neutral, 2-Disagree, 1-Strongly Disagree). The reliability of this scale is discussed in the results section of this study.

#### 3.3.2 Helpfulness of AI application courses scale

The Helpfulness of AI Application Courses Scale was adapted from Han (2023) and includes five subscales (21 items in total): course objectives (4 items), course resources (5 items), evaluations (4 items), technological assistance (4 items), and online and in-class activities (4 items). The scale employs a five-point Likert scale (5-Strongly Agree, 4-Agree, 3-Neutral, 2-Disagree, 1-Strongly Disagree). Han’s (2023) research indicates that the scale possesses robust reliability and validity. The internal reliability coefficient was 0.949. Alpha reliabilities for the subscales were 0.873, 0.877, 0.910, 0.902, and 0.881, respectively, with each subscale’s Average Variance Extracted (AVE) exceeding 0.50, suggesting strong reliability and convergent validity. Composite reliability (CR) values exceeded 0.80, demonstrating high reliability. Exploratory and Confirmatory Factor Analyses confirmed the scale’s high construct validity and adequate discriminant validity.

### 3.4 Data collection, analysis, and presentation

Data collection received prior approval from the Institutional Review Board (IRB). Informed consent provided participants with details about the study’s purpose, the estimated time required for the survey, potential challenges and benefits, researcher contact information, and other relevant details. Participants were informed that their participation was voluntary and that their responses would be anonymous. Data were collected at the end of June 2024, strategically timed after students had completed all five AI courses.

To address RQ1, descriptive statistics (mean, standard deviation, and mean of each item) were reported for the pre-service teachers’ level of AI competency overall and for each subscale (AI understanding and awareness, basic AI knowledge, basic AI skills, problem-solving, AI teaching practice, and ethics and safety). The study then employed an independent samples *t*-test to compare AI competency scores between students who had not participated in AI courses (pre-test group) and those who had completed all five AI courses (post-test group). If the post-course group scores significantly higher, it preliminarily suggests that the AI courses may have enhanced teacher competency.

To address RQ2, descriptive statistics (mean, standard deviation, minimum rating, and maximum rating) were calculated for pre-service teachers’ perspectives on the overall scores and subscales of the measure of AI course helpfulness (Introduction to AI technology, AI programming language, AI education technology, Teaching AI to primary school students, and Interdisciplinary teaching applications of AI for primary school students). Pearson correlations were then calculated between key variables (AI competency and perceptions of helpfulness of AI courses) and control variables (gender and teaching subject). If the correlation results are significant, it demonstrates that the perceived helpfulness of AI courses is significantly correlated with teacher AI competency. To further explore the influence of perceived helpfulness, post-test group was further divided into high- and low-perception subgroups based on the median score of perceived course helpfulness. An independent samples *t*-test was used to compare the differences in AI competency between subgroups with high and low perceptions of course helpfulness.

RQ3 investigates the relationship between pre-service teachers’ perceptions of AI course helpfulness and their AI competencies. Pre-service teachers’ AI competencies were regressed on their perceptions of the helpfulness of AI courses to explore the association between perceived course usefulness and AI competencies.

## 4 Results

### 4.1 RQ1: pre-service teachers’ AI competency difference between pre-test and post-test groups

A descriptive statistical analysis was conducted on the pre-test and post-test scores for overall and subscales of AI competency among 79 participants, comparing the pre-test group who had not completed any courses with the post-test group who completed all five courses (See Table 3). Higher scores indicated that the pre-service teachers perceived higher AI competency, with responses ranging from 1 to 5, representing “Strongly Disagree,” “Disagree,” “Neutral,” “Agree,” and “Strongly Agree,” respectively. Before the participants undertook the AI courses, the lowest mean scores were observed in basic AI knowledge (M = 3.01), problem-solving (M = 3.18), and basic AI skills (M = 3.32). The highest mean scores were in AI awareness (M = 3.53), AI teaching practice (M = 3.41), and ethics and safety (M = 3.49). Based on the calculation of Cohen’s d, basic AI knowledge and total AI competency exhibited a large effect size. AI awareness and basic AI skills approached a large effect size. The remaining dimensions showed small to medium effect sizes. Furthermore, the mean total scores of AI competency indicated that, on average, pre-service teachers viewed their AI competency as having significantly increased after competing all five AI courses. The results also demonstrated that the post-test group exhibited higher AI competency across all subscales compared to the pre-test group. Specifically, AI awareness (M = 4.03), basic AI knowledge (M = 3.77), and basic AI skills (M = 3.74) showed notable improvements. The results also demonstrated that the post-test group exhibited higher AI competency across all subscales relative to the pre-test group.

**TABLE 3:** Descriptive statistics for pre-test and post-test groups on overall scores and subscales of artificial intelligence (AI) courses.

Variable	Reliability	Number of course completed	Mean of total scores	Mean of each item	SD	Std. error mean	Cohen’s *d*
AI awareness	/	0	10.59	3.53	1.62	0.1824	0.85
	/	5	12.08	4.03	1.89	0.2133	
Basic AI knowledge	/	0	9.02	3.01	2.15	0.2423	1.08
	/	5	11.32	3.77	2.09	0.2354	
Basic AI skills	/	0	9.96	3.32	1.78	0.2009	0.73
	/	5	11.24	3.74	1.73	0.1950	
Problem Solving	/	0	9.54	3.18	1.83	0.2068	0.42
	/	5	10.36	3.45	2.05	0.2308	
AI teaching practice	/	0	10.24	3.41	1.65	0.1865	0.39
	/	5	10.93	3.64	1.91	0.2149	
Ethics and safety	/	0	10.48	3.49	1.78	0.2005	0.49
	/	5	11.36	3.78	1.80	0.2031	
Total AI competency	0.843	0	59.58	3.31	8.09	0.9112	0.91
	0.865	5	67.32	3.74	8.87	0.9989	

To further explore the differences in scores between the two groups, an independent samples *t*-test was conducted. Table 4 below presents the results of the *t*-tests comparing the means of AI competency subscales and total scores between the two groups. Levene’s Test for Equality of Variances was employed to assess the homogeneity of variances, and *t*-tests were conducted to determine if there were significant differences between the groups.

**TABLE 4:** Independent samples *T*-test.

Variable		Levene’s test for equality of variances		*t*-test for equality of means					95% confidence interval of the difference	
		*F*	Sig.	t	df	Sig. (2-tailed)	Mean difference	Std. error difference	Lower	Upper
AI awareness	Equal variances assumed	0.429	0.513	−5.322	156	0.000	−1.49367	0.28067	−2.04808	−0.93927
	Equal variances not assumed	–	–	−5.322	152.330	0.000	−1.49367	0.28067	−2.04818	−0.93916
Basic AI knowledge	Equal variances assumed	1.206	0.274	−6.819	156	0.000	−2.30380	0.33786	−2.97117	−1.63642
	Equal variances not assumed	–	–	−6.819	155.868	0.000	−2.30380	0.33786	−2.97117	−1.63642
Basic AI skills	Equal variances assumed	0.029	0.864	−5.515	156	0.000	−1.54430	0.28001	−2.09741	−0.99120
	Equal variances not assumed	–	–	−5.515	155.864	0.000	−1.54430	0.28001	−2.09741	−0.99120
Problem solving	Equal variances assumed	2.463	0.119	−2.655	156	0.009	−0.82278	0.30994	−1.43501	−0.21056
	Equal variances not assumed	–	–	−2.655	154.155	0.009	−0.82278	0.30994	−1.43507	−0.21050
AI teaching practice	Equal variances assumed	2.577	0.110	−2.446	156	0.016	−0.69620	0.28460	−1.25837	−0.13403
	Equal variances not assumed	–	–	−2.446	152.969	0.016	−0.69620	0.28460	−1.25846	−0.13394
Ethics and safety	Equal variances assumed	0.081	0.776	−3.104	156	0.002	−0.88608	0.28545	−1.44991	−0.32224
	Equal variances not assumed	–	–	−3.104	155.973	0.002	−0.88608	0.28545	−1.44991	−0.32224
Total AI competency	Equal variances assumed	0.620	0.432	−5.732	156	0.000	−7.74684	1.35147	−10.41637	−5.07730
	Equal variances not assumed	–	–	−5.732	154.729	0.000	−7.74684	1.35147	−10.41654	−5.07713

For AI awareness, the results indicated no significance difference in variances between the groups, *F* = 0.429, *p* = 0.513 > 0.05. The *t*-test results showed that, assuming equal variance, there was no significant difference in AI awareness between pre-test and post-test groups t(156) = −5.32, *p* < 0.001, 95% CI = (−2.04808, −0.93927). Therefore, the null hypothesis was rejected, indicating there was sufficient evidence to conclude that the mean AI awareness scores differ significantly between pre-test and post-test groups. These results suggested that the AI courses impacted on pre-service teachers’ AI awareness. Similarly, for basic AI knowledge between two groups, Levene’s Test for equal variance showed *F* = 1.206, *p* = 0.274 > 0.05, indicating no significant variance difference. Assuming equal variance, the *t*-test result was t(156) = −6.819, *p* < 0.001, 95% CI = (−2.97117, −1.63642). The null hypothesis was rejected, suggesting that the AI courses had a significant impact on pre-service teachers’ basic AI knowledge. Basic AI skills also showed a significant difference between two groups. Levene’s Test for equal variance showed *F* = 0.029, *p* = 0.864 > 0.05, indicating equal variances. The *t*-test result, assuming equal variance, was t(156) = −5.515, *p* < 0.001, 95% CI = (−2.09741, −0.99120). Therefore, the null hypothesis was rejected, indicating the AI courses significantly affected pre-service teachers’ basic AI skills. In terms of problem solving ability, Levene’s Test for equal variance showed *F* = 2.463, *p* = 0.119 > 0.05, indicating no significant variance difference. Assuming equal variance, t(156) = −2.655, *p* = 0.009, 95% CI = (−1.43501, −0.21056). Therefore, the null hypothesis was rejected, showing that the AI courses had a significant impact on pre-service teachers’ problem solving ability. For AI teaching practice, Levene’s Test for equal variance showed *F* = 2.577, *p* = 0.110 > 0.05, indicating no significant variance difference. Assuming equal variance, t(156) = −2.446, *p* = 0.016, 95% CI = (−1.25837, −0.13403). Therefore, the null hypothesis was rejected, suggesting that the AI courses had a significant impact on pre-service teachers’ AI teaching practice. Regarding ethics and safety, Levene’s Test for equal variance showed *F* = 0.081, *p* = 0.776 > 0.05, indicating equal variances. The *t*-test result, assuming equal variance, was t(156) = −3.104, *p* = 0.002, 95% CI = (−1.44991, −0.32224). The null hypothesis was rejected, meaning the AI courses significantly affected pre-service teachers’ awareness of ethics and safety in AI. For the total AI competency score, Levene’s Test for equal variance showed *F* = 0.620, *p* = 0.432 > 0.05, indicating no significant variance difference. Assuming equal variance, t(156) = −5.732, *p* < 0.001, 95% CI = (−10.41637, −5.07730). The null hypothesis was rejected. The results indicated that the AI courses exerted a significant impact on pre-service teachers’ overall AI competency.

Therefore, the results of the independent samples *t*-tests across all variables (AI awareness, basic AI knowledge, basic AI skills, problem-solving, AI teaching practice, ethics and safety, and total AI competency) indicate that the overall and subscale scores of AI competency differed significantly between the two groups. In all cases, the null hypothesis of no difference between the pre-test and post-test groups was rejected. To directly answer Research Question 1, there is a significant difference in teacher AI competency between the pre-test group and the post-test group.

### 4.2 RQ2: associations between AI course helpfulness and teacher AI competency

RQ2 explored the associations between AI course helpfulness and pre-service teachers’ AI competency. Table 5 displayed descriptive statistics for the perceived helpfulness of AI courses, which included objectives, activities, resources, assessments, and technology support for overall courses and each course, respectively. High scores indicate that pre-service teachers perceive the course as more helpful, with responses ranging from “Strongly Agree” to “Strongly Disagree.” Overall, the mean score of 96.39 suggested that pre-service teachers generally found the AI courses to be “helpful.” They perceived “Teaching AI for primary school students” (mean = 19.49) and “Interdisciplinary teaching applications of AI for primary school students” (mean = 19.31) as the most helpful, while AI Education Technology was seen as the least helpful (mean = 19.07). Thus, the results revealed that pre-service teachers perceived the AI courses as helpful.

**TABLE 5:** Descriptive statistics for the overall scores and subscales of the measure of artificial intelligence (AI) course helpfulness (*N* = 79).

Variable	Reliability	Numbers of items	Min	Max	Mean	SD
Overall AI courses helpfulness	0.872	21	64.00	125.00	96.39	14.00
Overall “*Introduction to Artificial Intelligence Technology*” helpfulness	0.890	21	13.00	25.00	19.24	2.89
Objectives	–	4	2.00	5.00	3.82	0.65
Activities	–	5	2.00	5.00	3.82	0.71
Resources	–	4	3.00	5.00	3.89	0.67
Assessment	–	4	2.00	5.00	3.83	0.72
Technology support	–	4	3.00	5.00	3.86	0.59
Overall “*AI programming language*” helpfulness	0.832	21	13.00	25.00	19.26	2.89
Objectives	–	4	2.00	5.00	3.84	0.68
Activities	–	5	3.00	5.00	3.92	0.61
Resources	–	4	1.00	5.00	3.83	0.70
Assessment	–	4	2.00	5.00	3.79	0.72
Technology support	–	4	2.00	5.00	3.86	0.69
Overall “*AI Education Technology*” helpfulness	0.859	21	10.00	25.00	19.07	3.29
Objectives	–	4	2.00	5.00	3.82	0.74
Activities	–	5	2.00	5.00	3.88	0.73
Resources	–	4	2.00	5.00	3.74	0.74
Assessment	–	4	2.00	5.00	3.84	0.71
Technology support	–	4	2.00	5.00	3.77	0.79
Overall “*Teaching AI for primary school student*” helpfulness	0.865	21	13.00	25.00	19.49	2.99
Objectives	–	4	2.00	5.00	3.92	0.67
Activities	–	5	2.00	5.00	3.91	0.64
Resources	–	4	2.00	5.00	3.84	0.80
Assessment	–	4	2.00	5.00	3.89	0.67
Technology support	–	4	3.00	5.00	3.91	0.68
Overall “*Interdisciplinary teaching applications of AI for primary school students*” helpfulness	0.870	21	13.00	25.00	19.31	2.89
Objectives	–	4	3.00	5.00	3.89	0.61
Activities	–	5	1.00	5.00	3.81	0.83
Resources	–	4	2.00	5.00	3.91	0.71
Assessment	–	4	1.00	5.00	3.81	0.80
Technology support	–	4	2.00	5.00	3.88	0.65

Pearson correlations were examined among key variables (AI competency and AI course helpfulness) and control variables (gender and subject). Table 6 indicated that perceived helpfulness of AI courses was significantly correlated with pre-service teachers’ AI competency (*r* = 0.511, *p* < 0.01). However, no significant correlations were found between gender or subject and either perceived course helpfulness or AI competency (i.e., gender and subject did not correlate with these variables).

**TABLE 6:** Descriptive statistics and Pearson correlation between key variables in the regression models with control variables (*N* = 79).

	Correlations		
Variables	2	3	4
1. Gender (1 = female)	−0.202	−0.103	0.806
2. Subject (1 = math/science)	–	−0.031	−0.150
3. Overall AI competency of post-test group	–	–	0.511**
4. Course helpfulness	–	–	–

**P < 0.01.

The study also conducted a linear regression analysis to explore the predictive effect of the helpfulness of AI courses on pre-service teachers’ AI competencies. The output showed that the coefficient of determination R^2^ is 0.261. This value indicates that approximately 26.1% of the variance in the dependent variable can be explained by the independent variable. In this context, the value of 0.261 reflected a moderate practical significance of the regression model, meaning the helpfulness of AI courses has a noticeable predictive relationship with pre-service teachers’ AI competencies.

To further explore whether the perception of helpfulness of AI courses influenced pre-service teachers’ AI competency, post-test group was divided into low- and high-perception subgroups based on the median score of course helpfulness (*M* = 96.39). Table 7 indicated that there were 38 cases in the low-perception group and 41 in the high-perception group, with mean scores of 63.00 (SD = 0.920) and 71.34 (SD = 1.476), respectively.

**TABLE 7:** Descriptive analysis for high artificial intelligence (AI)-course helpfulness group and low AI-course helpfulness groups.

Variable	Group	Case number	Mean	Standard error
AI competency after taking AI courses	Low-perception of course helpfulness	38	63.00	0.920
	High-perception of course helpfulness	41	71.34	1.476

An independent samples *T*-test was conducted to compare the differences in AI competency between the high-perception and low-perception subgroups. The results in Table 8 showed significant differences in variances between the groups, *F* = 8.902, *p* = 0.004 < 0.05. The *t*-test results indicated a statistically significant difference in AI competency scores between the two groups, t(66.328) = −4.795, *p* < 0.001, 95% CI = (−11.81458, −4.86834). Therefore, the null hypothesis was rejected, providing substantial evidence that pre-service teachers in the high AI-course helpfulness group had significantly higher AI competency than those in the low helpfulness group. These results suggested that perceived course helpfulness significantly influenced AI competency development, highlighting the importance of effective AI course design for educators. The results suggested that perceived course helpfulness exerted a significant influence on the development of pre-service teachers’ AI competency. This underscored the importance of effective AI course design for educators.

**TABLE 8:** Independent samples *T*-test on artificial intelligence (AI) competency scores between low-perception of course helpfulness group and high-perception of course helpfulness groups.

Variable		Levene’s test for equality of variances		*t*-test for equality of means					95% confidence interval of the difference	
		*F*	Sig.	t	df	Sig. (2-tailed)	Mean difference	Std. error difference	Lower	Upper
AI competency	Equal variances assumed	8.902	0.004	−4.709	77	0.000	−8.34146	1.77144	−11.86885	−4.81408
	Equal variances not assumed	–	–	−4.795	66.328	0.000	−8.34146	1.73971	−11.81458	−4.86834

### 4.3 RQ3: predicting pre-service teachers’ overall AI competency from AI course helpfulness

RQ3 was designed to examine the association between pre-service teachers’ overall AI competency and perceptions of the helpfulness of AI application courses after controlling for gender and teaching subjects. It aimed to test the influence of perceptions of the helpfulness of AI courses on pre-service teachers’ overall AI competency after controlling for gender and teaching subjects.

The overall AI competency scores were regressed on the total ratings pre-service teachers gave regarding the helpfulness of AI application courses, including the helpfulness of Introduction to AI Technology, AI programming language, AI Education Technology, Teaching AI for primary school students, and Interdisciplinary teaching applications of AI for primary school students. The full model was statistically significant, F(3, 75) = 9.883, *p* < 0.01, with gender, major, and AI course helpfulness ratings all accounting for statistically significant proportions of unique variation in AI competency (Table 9). Among control variables, neither gender nor subject significantly predicted AI competency. Directly addressing RQ3, an increase of one point in AI-course helpfulness rating was associated with a.333 point increase in teacher AI competency (*b* = 0.333, *p* < 0.001). Therefore, directly addressing RQ3, a one-point increase in the AI course helpfulness rating was associated with a.333-point increase in teachers’ AI competency.

**TABLE 9:** Summary of simultaneous multiple linear regression results predicting pre-service teachers’ overall artificial intelligence (AI) competency from perceptions of the helpfulness of AI application courses.

	*b*	*SE*_b_	β	t	*P*
**Control variables**					
Gender (0 = male)	–	–	–	–	–
Female (1 = female)	−2.985	2.062	−0.145	−1.448	0.152
Subject (0 = other subjects)	–	–	–	–	–
Math or science (1 = math/science)	0.352	1.857	0.019	0.190	0.850
**Predictor variable**					
Helpfulness of courses	0.333	0.063	0.526	5.314	0.000**

**P < 0.01. *R* = 0.532, *R*^2^ = 0.283, F(3, 75) = 9.883, *p* < 0.01.

## 5 Discussion

### 5.1 Current level of pre-service teachers’ AI competency

RQ1 indicated that the overall and subscale levels of AI awareness, basic AI knowledge, basic AI skills, AI teaching practice, problem-solving, and ethics and safety among pre-service teachers were at a moderate level.

This finding aligns with the literature, which underscores that AI competency is a relatively new development, suggesting significant potential for further exploration by both researchers and educators in this emerging field. Previous studies have explored the current levels of AI competency among pre-service teachers. For example, Ayanwale et al. (2024) surveyed 529 pre-service teachers and, along with other researchers, recommended that they become familiar with AI-enhanced technologies, including basic AI knowledge and skills such as adaptive educational systems, intelligent tutors, and automatic feedback (Ayanwale et al., 2022; Gunkel, 2012; Han, 2021; Huang, 2021). It also emphasized the need to recognize ethical considerations (Akgun and Greenhow, 2021; Hayes and Kraemer, 2017; Long and Magerko, 2020) and responsible AI use (Alfalah, 2018; Lin et al., 2023), as noted in other studies. Similarly, Vazhayil et al. (2019) assessed 34 pre-service teachers, highlighting that challenges in AI competency were related to teaching, designing appropriate methods, and selecting age-appropriate materials. It indicated that pre-service teachers were not well-prepared to integrate AI teaching practices into daily classroom instruction (Frimpong, 2022; Ng et al., 2021; Su et al., 2022; Wong et al., 2020). Additionally, despite increasing efforts in policy and resource development to popularize AI technology in education, previous work demonstrated minimal evidence supporting professional development programs and supportive AI courses for both in-service and pre-service teachers (Mike and Rosenberg-Kima, 2021; Lee and Perret, 2022; Sanusi, 2021). Therefore, the moderate level of AI competency among pre-service teachers reflects a transitional phase in which AI is being gradually introduced into educational settings, with foundational understanding yet requiring further development in practical application and advanced skills. This also underscores the importance of continued efforts to strengthen AI competency through targeted curriculum enhancements, professional development opportunities, and the creation of supportive learning environments.

### 5.2 Impact of AI course participation on competency gains

Results of the study found that pre-service teachers who attended AI courses (post-test group) had higher AI competency across all measured areas compared to those who had not participated in any AI courses (pre-test group). Specifically, notable improvements were observed in basic AI knowledge, AI awareness, and basic AI skills. The finding that participation in structured AI courses significantly improves partial AI competency supports Ayanwale et al.’s (2024) critical observation that mere exposure to AI tools may not enhance educators’ ability to recognize AI implementations, while systematic training that integrates theoretical knowledge with contextualized learning holds promise for bridging this gap, though its effectiveness may vary depending on course design, learners’ prior experience, and opportunities for practical application. This discrepancy indicates that fragmented technological exposure alone is inadequate (Mhlongo et al., 2023); rather, intentional pedagogical scaffolding promotes deeper cognitive engagement, enabling teachers to move beyond superficial tool usage to a more critical awareness and competency development (Alam and Mohanty, 2023; Al-khresheh, 2024; Bearman and Ajjawi, 2023).

Courses such as *Introduction to AI Technology* comprehensively covered the technical principles, such as Python-based facial recognition, of core AI domains including machine learning, computer vision, and natural language processing. Knowledge about AI and learning about subjective norms directly strengthened pre-service teachers’ systematic mastery of fundamental AI knowledge (Habibi et al., 2022). The importance of basic AI knowledge and the subjective norm as key factors stimulating pre-service teachers’ intention to learn AI was also demonstrated in a study by Sanusi et al. (2024), which surveyed 796 pre-service teachers and found that “basic AI knowledge and subjective norm influence pre-service teachers’ realization of personal relevance of learning AI” (p.10). Moreover, the *AI programming language* course was specifically designed to develop students’ basic AI knowledge and basic AI skills by introducing foundational programming languages such as Python and Scratch, along with other AI tools widely used in primary education. Through a structured curriculum, students gained a solid understanding of core AI concepts, including the fundamentals of machine learning, algorithmic thinking, and the application of AI technologies in educational settings. Practical exercises and hands-on projects were integral components of the course, allowing students to develop essential programming skills and apply them to real-world scenarios. This approach ensured that students were well-prepared to effectively leverage AI tools and adapted them to diverse teaching and learning environments (Chen et al., 2022). As such, pre-service teachers recognized that acquiring foundational AI knowledge and basic AI skills was highly relevant to their future professional effectiveness, thereby enhancing their AI awareness (Li et al., 2022).

### 5.3 Challenges in problem-solving and teaching practice competencies

The study revealed that pre-service teachers had less improvement in AI problem-solving and AI teaching practice compared to other competencies, despite their perception of greater helpfulness from AI courses such as *Interdisciplinary Teaching Applications* and *Teaching AI for primary school students*.

The primary reason was that AI problem-solving competencies require integrating higher-order cognitive skills and synthesizing multiple AI concepts. Walter (2024) noted that the transformative impact of AI in educational settings should emphasize skills such as AI literacy, prompt engineering proficiency, and enhanced critical thinking. AI literacy encompasses an understanding of AI technologies and their broader societal impacts (Casal-Otero et al., 2023). Prompt engineering was identified as a crucial skill for generating tailored responses from AI systems, thereby enhancing educational experiences and fostering critical thinking (Lee, 2022). These advanced skills are inherently more complex to develop compared to foundational knowledge or basic skills, often requiring extended time, iterative practice, and structured scaffolding to achieve proficiency (Chan, 2023; Chiu et al., 2023).

Moreover, improving AI teaching practice competency requires not only technical proficiency but also the effective application of pedagogical strategies (Ayanwale et al., 2024; Kim et al., 2022). Previous research has indicated that pre-service teachers face challenges when translating AI concepts into classroom settings. For instance, Carvalho et al. (2022) highlighted that teachers may encounter difficulties integrating AI into practical tasks such as automated assessment and evaluation, despite recognizing its benefits. Filiz et al. (2025) pointed out that barriers for teachers included adapting AI to distinct educational contexts (i.e., designing age-appropriate AI activities for primary school students) and curricular misalignment (i.e., adapting AI-generated content to local contexts), often due to limited practical exposure to real-world teaching environments or inadequate guidance on aligning AI integration with existing curricula. This finding aligns with the conceptual distinction between AI literacy and competency: while literacy (e.g., understanding AI basics) can be developed through foundational courses, competency—especially its expert judgment component (e.g., adapting AI to classroom contexts or solving interdisciplinary problems)—requires more intensive practice and contextualized learning, which were insufficient in the current curriculum design.

Furthermore, constraints in course design may have contributed to the limited improvement in these areas. As outlined in Table 2, the curriculum allocated a larger proportion of credits (2 out of 5 courses) to foundational knowledge (e.g., *Introduction to AI Technology, AI Programming Language*) compared to problem-solving and teaching practice (1 course each). While courses such as Interdisciplinary Teaching Applications aimed to address practical integration, they focused more on theoretical case analysis than hands-on practice in real classroom settings—such as designing and implementing AI-integrated lessons in actual primary school classrooms. Additionally, higher-order AI concepts critical for problem-solving (e.g., machine learning logic, adaptive learning system design) were not sufficiently embedded in the curriculum, which misaligns with the worldwide trend of integrating genuine higher-order AI concepts into teacher education (Walter, 2024; Casal-Otero et al., 2023). This gap between course design and the demand for advanced, contextually applied skills further hindered the development of problem-solving and teaching practice competencies. Besides, the improvement of AI teaching practice competency may be hindered by insufficient integration of ethical and sociopolitical considerations in course design. While pre-service teachers reported moderate gains in basic ethics awareness (Table 3), courses did not adequately address complex scenarios such as identifying algorithmic bias in AI-generated lesson plans, balancing personalized learning with student data privacy, or navigating tensions between global AI tools and local digital sovereignty (Filiz et al., 2025; Walter, 2024). Without training in these areas, teachers may lack confidence in applying AI ethically in real classrooms, contributing to the observed weakness in teaching practice competency. Therefore, it is reasonable that pre-service teachers exhibited relatively lower competencies in problem-solving and AI teaching practice.

### 5.4 Factors associated with AI competency

RQ3 demonstrated a positive association between the perceived helpfulness of AI courses and pre-service teachers’ AI competencies. Moreover, RQ3 results indicated that neither gender nor teaching subjects significantly predicted teachers’ AI competency. This finding contrasted with Chiu and Churchill’s (2016) study, which showed that science and mathematics teachers held more positive attitudes toward technology compared to language and humanities teachers. However, more recent literature supports the present study’s findings. For example, Darayseh (2023) studied a sample of 83 teachers in Abu Dhabi and found no statistically significant differences in teacher responses regarding their intentions to use AI in science teaching, based on variables such as gender, teaching experience, and qualifications. This phenomenon could be attributed to teachers’ comparable competencies and situations, resulting in minimal differentiation among them. This lack of gender-based differences in AI competency might reflect the equalizing potential of structured and useful AI coursework, whereby both male and female pre-service teachers could acquire foundational AI skills independently of prior technology experience or interests—though such coursework must also address potential risks, such as over-reliance on technology or uneven access to learning resources. This result also aligned with the findings of Almousa (2020), indicating no statistically significant variations based on experience levels among female teachers’ intentions to utilize AI technologies in classroom instruction.

## 6 Limitations and suggestions for future research

Whereas this study provides insights into the relationship between the perceived helpfulness of AI courses and AI competency, several limitations should be acknowledged. Firstly, the study relied on self-reported data. It may be susceptible to social desirable bias, where participants might overstate their perceived competency or the helpfulness of courses to align with positive expectations of AI education interventions. This could potentially lead to over-reporting of the intervention effect, which should be acknowledged as a limitation. Future research could utilize data triangulation to integrate multiple data sources, such as classroom observations of pre-service teachers’ AI application practices, qualitative interviews on their teaching experiences, and performance-based assessments (e.g., AI-integrated lesson plans), to cross-validate reported competencies with actual teaching behaviors. Additionally, longitudinal follow-up studies could be conducted to track pre-service teachers’ AI competencies over an extended period (e.g., 1–2 years after graduation), examining the sustainability of observed improvements and their practical application in long-term teaching practices.

Secondly, the quasi experimental design employed in this study, while effective for comparing changes in competencies before and after AI courses, is potentially constrained by confounding variables—such as external learning experiences beyond the scope of the courses—that may have influenced the observed improvements. Future research would benefit from adopting more rigorous methodologies, such as controlled trials incorporating a comparison group, to more precisely isolate the specific effects of AI courses. Furthermore, the convenience sample was derived from a single university in Shanghai, which may introduce homogeneity in areas such as curriculum design, educational resources, and regional educational policies. This limits the generalizability of the findings to pre-service teachers from other institutions, regions, or educational systems (e.g., comprehensive universities or institutions in other provinces). Additionally, the over-representation of female participants (82.2%) and language arts majors (67.1%) may limit the generalizability of findings to more gender-balanced samples or pre-service teachers in STEM fields, as prior studies suggest potential variations in technology engagement across genders and subjects (Chiu and Churchill, 2016). Future research should aim for a more balanced demographic distribution to verify whether the observed patterns are consistent across diverse groups.

Thirdly, the current study relied exclusively on quantitative self-report data, which precluded nuanced insights into how and why specific course modules (e.g., practical programming exercises or interdisciplinary teaching cases) influence pre-service teachers’ perceived self-efficacy in AI application. Notably, no qualitative inputs (e.g., interviews or reflective journals) were included to unpack the contextual mechanisms that link course design to self-efficacy. Furthermore, the study failed to account for mediating or moderating variables identified in the literature as critical to technology adoption (Ayanwale et al., 2022)—for instance, prior technology experience or attitudes toward AI. Such variables may moderate or mediate the relationship between course helpfulness and AI competency, thereby limiting our understanding of the complex dynamics underlying competency development. To address these gaps, future research should adopt a mixed-methods design that integrates qualitative data, employ advanced analytical frameworks (e.g., structural equation modeling) to examine moderating and mediating effects, expand to larger and more diverse samples across multiple institutions, and incorporate longitudinal tracking to assess the long-term retention and classroom application of AI competencies.

In conclusion, this study highlights the critical role of perceived AI course helpfulness in shaping pre-service teachers’ AI competencies. Expanding AI education within teacher preparation programs, when designed to balance technical skills, ethical awareness, and practical application, can better equip educators to navigate the evolving landscape of AI-enhanced teaching and learning environments. This approach, however, must be paired with a recognition of the need for sustained support systems to address barriers to implementation.

## 7 Conclusion

This research aimed to examine pre-service teachers’ perceptions of their AI competency and determine how participation in AI courses affects these perceptions and actual competency levels. Additionally, this study examined perceptions regarding the helpfulness of AI courses in enhancing pre-service teachers’ AI competencies and explored how these perceptions influence their actual AI competency. In addressing these questions, the findings indicated that participation in AI courses enhanced both pre-service teachers’ ability to impart AI skills and their confidence levels.

These findings have several important implications for educational sustainability and teacher training programs, specifically for national curriculum developers, teacher educators, and educational policymakers. Firstly, the results highlight the importance of integrating AI courses into current teacher training curricula. As AI is increasingly integrated into education, it is essential that teachers possess AI knowledge and the ability to apply relevant AI tools and practices effectively in their classrooms. Consequently, teacher education programs should emphasize course content relevance and perceived helpfulness, ensuring pre-service teachers find these courses beneficial for their future teaching practices. The second recommendation is to offer more AI-related courses across diverse subject areas. The significant improvement in competencies associated with multiple AI courses suggests that a single introductory AI course might not sufficiently prepare pre-service teachers. Instead, AI courses should be widely integrated into teacher education programs, covering various aspects ranging from programming skills and ethical considerations to preparing educators for the complexities of teaching in AI-enhanced classrooms. Third, to address the ethical and sociopolitical dimensions of AI in education—now a central focus of global discourse—courses should integrate targeted modules on identifying algorithmic bias, establishing ethical boundaries for classroom surveillance, and advancing digital sovereignty in data management. Such integration will equip pre-service teachers to navigate not only technical challenges but also the equity and autonomy concerns that underpin responsible AI integration across diverse educational contexts (UNESCO, 2024; Akgun and Greenhow, 2021). Finally, the findings suggest the potential for interdisciplinary integration of AI education. Since liberal arts and STEM teachers showed no significant differences in AI competencies, it appears that AI competencies are becoming relevant across all subject areas. This supports the development of interdisciplinary AI courses, allowing all educators to effectively integrate AI into their teaching practices.

## Data Availability

The original contributions presented in this study are included in this article/supplementary material, further inquiries can be directed to the corresponding author.
